# Resistant *Escherichia coli *strains circulating in a tertiary-care hospital in New Delhi, India

**DOI:** 10.1186/cc11769

**Published:** 2012-11-14

**Authors:** B Uppal, P Aggarwal, R Ghosh, AK Jha, S Krishna Prakash

**Affiliations:** 1 Maulana Azad Medical College, New Delhi, India

## Background

*Escherichia coli *is a leading cause of bloodstream infections worldwide and its associated rate of mortality is high. It is found in its primary habitat, the digestive tract, as a commensal but is also involved in various intestinal and extraintestinal diseases. It is a known fact that the digestive tract may serve as a portal of entry into the bloodstream. Therefore, the presence of highly resistant *E. coli *strains in the gut presents a threat to the patients with predispositions such as chronic illnesses and poor immune status.

## Methods

This study was undertaken with the aim to determine the resistance pattern among the *E. coli *strains isolated from the gut of chronically ill patients across wide clinical settings. The study was conducted over a period of 1 year from 1 January 2011 to 31 December 2011. Stool samples from patients admitted for more than 14 days in wards of all major clinical specialities were collected after proper counselling and informed consent. *E. coli *were identified on the basis of cultural characteristics and biochemical reactions. Strains isolated from pediatric patients were subjected to serotyping by the slide agglutination test with specific antisera (Denka Seiken Co., Ltd, Tokyo, Japan) to identify the enterovirulent strains. All *E. coli *strains were subjected to antimicrobial susceptibility testing and ESBL identification by the disk diffusion methods in accordance with the CLSI guidelines.

## Results

Two hundred and fifty-four patients were included in the study with the following distribution: 71 from a pediatrics ward, 62 from a general medicine ward, 54 from a general surgery ward, 42 from a gynaecology ward, 16 from an orthopaedics ward and nine from an ENT ward. *E. coli *was isolated from 112 samples. Out of 34 *E. coli *strains isolated from paediatric patients, 14 were determined to be enterovirulent *E. coli *by serotyping. Antimicrobial susceptibility testing of all *E. coli *strains showed 100% resistance to nalidixic acid and a high degree of resistance to ampicillin (87.5%), doxycyline (83.0%), cotrimoxazole (75.9%), ciprofloxacin (73.2%) and third-generation cephalosporins (71.4%). ESBL production was detected in 71 strains (63.4%). However, no resistance was found for carbapenems and tigecycline.

## Conclusion

A large population of chronically ill patients who were tested was found to be carrying highly resistant *E. coli *in their guts. These usually commensal strains may serve as a source of bloodstream infection especially in cases of immunosuppression. Whether these strains were acquired in the hospital or from the community needs to be studied further.

